# Anomalous Precipitation of the γ-Fe Phase in Fe-Based Nanocrystalline Alloys and Its Impact on Soft Magnetic Properties

**DOI:** 10.3390/ma18122867

**Published:** 2025-06-17

**Authors:** You Wu, Lingxiang Shi, Ranbin Wang, Jili Jia, Wenhui Guo, Yunshuai Su, Hengtong Bu, Siqi Xiang, Weihong Yang, Mingli Fu, Yang Shao, Kefu Yao

**Affiliations:** 1 School of Materials Science and Engineering, Tsinghua University, Beijing 100084, China; wuyou21@mails.tsinghua.edu.cn (Y.W.); shilingxiang@bit.edu.cn (L.S.);; 2 School of Materials Science and Engineering, Beijing Institute of Technology, Beijing 100081, China; 3 China Southern Power Grid Electric Power Research Institute, Guangzhou 510663, China

**Keywords:** Fe-based nanocrystalline alloys, γ-Fe phase, soft magnetic properties, cooling rate, phase selection

## Abstract

High-Cu-content (Cu-content > 1.3 at.%) nanocrystalline alloys exhibit wide heat-treatment windows and favorable soft magnetic properties due to the presence of pre-existing α-Fe nanocrystals. By fabricating ribbons with varying thicknesses to tailor cooling rates, distinct structural characteristics were achieved in Fe_82_B_16.5_Cu_1.5_ alloy ribbons. Notably, the face-centered cubic (fcc) γ-Fe phase was identified in Fe-based nanocrystalline alloys. The precipitation of the fcc γ-Fe phase originates from a phase-selection mechanism under specific cooling conditions, while its retention in the as-quenched ribbon with a thickness of 27 μm is attributed to kinetic suppression during rapid cooling and the nanoscale stabilization effect. The formation of the fcc γ-Fe phase significantly reduced the saturation flux density (*B*_s_) and increased coercivity (*H*_c_), concurrently destabilizing the residual amorphous matrix. By suppressing the precipitation of the γ-Fe and Fe_3_B phases through precise control of ribbon thickness and annealing parameters, the alloy ribbon with a thickness of 16 μm achieved an optimal combination of *B*_s_ (1.82 T) and *H*_c_ (8.3 A/m). These findings on anomalous fcc γ-Fe phase precipitation provide novel insights into metastable phase engineering and offer structural design guidelines for alloys containing pre-existing α-Fe nanocrystals.

## 1. Introduction

Fe-based nanocrystalline alloys have gained widespread commercial preference, particularly in high-frequency applications (e.g., transformers and inductors) due to their low coercivity (*H*_c_), minimal core loss, and exceptional magnetic permeability [1,2,3]. These superior soft magnetic properties originate primarily from a dual-phase structure in which α-Fe nanocrystals (10–20 nm) embedded in an amorphous matrix collectively suppress magneto-crystalline anisotropy through exchange coupling [4,5]. A critical challenge to obtain such a desirable dual-phase structure lies in the precise control of nanocrystal evolution during annealing. Spontaneous abnormal coarsening of α-Fe grains during annealing severely deteriorates magnetic performance. Conventional stabilization strategies involve adding large atomic-sized elements (e.g., Nb, Zr, or Hf) to inhibit the rapid diffusion of Fe atoms, as exemplified by Fe-(Nb/Zr/Hf)-B alloys (NANOPERMs) [6,7,8]. However, the addition of these elements in large quantities degrades saturation flux density (*B*_s_) and introduces processing difficulty due to their high oxidation susceptibility. The commercially dominant Fe-Si-B-Cu-Nb alloy (FINEMET) achieves fine α-Fe nanograins through synergistic compositional design [9,10]. The positive mixing enthalpy between Fe and Cu drives the formation of Cu-rich clusters during annealing, which provide high-density nucleation sites, while Nb suppresses the growth kinetics of α-Fe nanograins [11]. This enables nanocrystal stabilization at low heating rates with prolonged holding time. Nevertheless, FINEMET’s moderate *B*_s_ (1.24 T) limits the power density when used in miniaturized high-frequency devices.

Recent breakthroughs made by Ohta and Zhang et al. demonstrate an alternative Nb-free approach using high Cu content (>1.3 at.%) [12,13,14,15,16,17]. High Cu promotes spontaneous fine α-Fe nanocrystal nucleation during rapid solidification. Kinetic competition between pre-existing and newly formed α-Fe nanocrystals during annealing limits the coarsening and a low *H*_c_ can be obtained under a low heating rate and long-time annealing [12,16]. The *B*_s_ of high-Cu-content nanocrystalline alloys reach 1.75–1.85 T by eliminating the dilution effect of Nb on *B*_s_ [12,14,17]. The *H*_c_ of high-Cu-content nanocrystalline alloys exhibits pronounced sensitivity to the as-quenched microstructure. Optimal soft magnetic properties are achieved when the as-spun ribbons contain a high density of ultrafine α-Fe nanocrystals [12]. The grain size and number density (*N*_d_) of pre-existing α-Fe nanocrystals are intrinsically correlated with the cooling rate during processing. However, inherent cooling rate gradients across the ribbon thickness—resulting from disparate heat transfer conditions between the free side and wheel side surface—induce significant variations in the α-Fe nanostructure within different regions of the as-quenched ribbon [18,19]. Such inhomogeneous structures along the thickness direction induce significant discrepancies in soft magnetic properties among ribbons with varying thicknesses [18]. To obtain the desirable size and distribution of α-Fe nanocrystals, a systematic study of the thicknesses on the structures of the as-quenched ribbon precursors is thus necessary. This study reveals not only structural differences in α-Fe across ribbons of varying thicknesses but also identifies the presence of the γ-Fe phase—typically stable only at elevated temperatures—in ribbons exceeding 21 μm. A comparative analysis demonstrates thickness-dependent phase composition, thermal stability, and soft magnetic properties, coupled with mechanistic investigations into γ-Fe phase precipitation. These findings provide critical guidance for microstructure design and performance optimization in high-Cu-content nanocrystalline alloys.

## 2. Methods

The Fe_82_B_16.5_Cu_1.5_ alloy ingots were prepared by induction melting under a high-purity argon atmosphere using pure Fe (99.98 mass%), B (99.5 mass%), and Cu (99.999 mass%). As-quenched Fe_82_B_16.5_Cu_1.5_ ribbons of various thicknesses were prepared under the argon atmosphere by the single-roller melt-spinning method with varying surface linear velocities of wheels (30–40 m/s). The ribbons with a thickness of 16 ± 1, 19 ± 1, 21 ± 1, and 27 ± 1 μm are denoted as T16, T19, T21, and T27, respectively. The thermal properties of these amorphous ribbons were measured by differential scanning calorimetry (DSC, Netzsch STA 449 F3, Selb, Germany) in a purified argon atmosphere at a heating rate of 40 K/min. The *B*_s_ and *H*_c_ were measured using a vibrating sample magnetometer (VSM, Lake Shore 7407 VSM, Lake Shore Cryotronics, Westerville, OH, USA) under the maximum applied field of 800 kA/m and a DC B-H loop tracer (MATS-2010SD, Linkjoin, Loudi, China) under a maximum applied field of 800 A/m. The structures of as-quenched and annealed samples were identified by X-ray diffraction (XRD, Rigaku D/max 2500, Tokyo, Japan) with a Cu Kα source and transmission electron microscopy (TEM, FEI Talos F200X, FEI, Hillsboro, OR, USA) equipped with energy-dispersive X-ray spectroscopy (EDS). TEM specimens near the middle layer and free side layer were prepared via double-sided and single-sided thinning methods, respectively, using an ion milling system (PIPS model 691, Gatan, Pleasanton, CA, USA) at 77 K. The average grain size (*D*_av_) of nanocrystals was determined through the fitting of the grain size distribution of nanocrystals within the TEM images. The *N*_d_ of nanocrystals was calculated by *N*_d_ = *N*/(*A* × *D*_av_), where N is the number of nanocrystals and A is the selected area of the TEM image [12].

## 3. Results and Discussion

### 3.1. Microstructure and Thermal Properties

**Figure 1:**
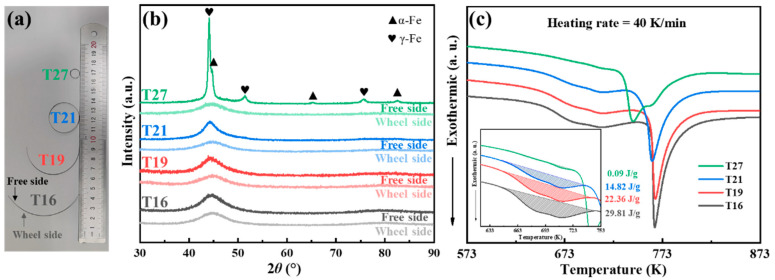
(**a**) Photo of the as-quenched Fe_82_B_16.5_Cu_1.5_ alloy ribbons with different thicknesses (the concave surfaces are the free sides); (**b**) XRD patterns of both surfaces of the as-quenched T16, T19, T21, and T27 samples; and (**c**) the DSC curves of the as-quenched T16, T19, T21, and T27 samples at a heating rate of 40 K/min. The inset shows the different crystallization enthalpy values at the first peak of the T16, T19, T21, and T27 samples.

T16, T19, T21, and T27 ribbons with thicknesses of 16 μm, 19 μm, 21 μm, and 27 μm were fabricated via the single-roller melt-spinning method. The average cooling rates, estimated using the method proposed by Lin and Johnson, were determined to be 3.9 × 10^6^ K/s, 2.8 × 10^6^ K/s, 2.3 × 10^6^ K/s, and 1.3 × 10^6^ K/s, respectively [20]. Interestingly, all the concave surfaces of T16–T27 are the free sides, as shown in Figure 1a. This implies that there may be some structural heterogeneity in the direction of ribbon thickness. Furthermore, with the increase in thickness, the curvature of alloy ribbons increases gradually. The elastic strain (*ε*) induced by structural heterogeneity can be expressed as *ε* = *t*/(2*r*_c_), where *t* is the ribbon thickness and *r*_c_ denotes the curvature radius of the ribbon. The T16, T19, T21, and T27 ribbons exhibited the *r*_c_ of 66.1 mm, 28.0 mm, 15.9 mm, and 5.0 mm, with the corresponding *ε* of 0.012%, 0.034%, 0.066%, and 0.27%, respectively. Amorphous materials undergo volumetric shrinkage during crystallization, leading to changes in density [21,22]. The variations in *ε* among T16–T27 may originate from structural inhomogeneity along the thickness direction: differences in volumetric shrinkage caused by variations in the degree of crystallinity. The XRD patterns were used to identify the structural differences between the free side and wheel side of T16–T27. As shown in Figure 1b, the broad diffraction peaks indicate the characteristic of amorphous structure in the T16–T27 wheel sides. In contrast, the broad diffractions begin to sharpen with the increase in ribbon thickness of the free side in T16, T19, and T21. For T27, the sharp diffractions matching the α-Fe and γ-Fe crystalline phases are found on the free side. The lattice parameter of the γ-Fe phase calculated from the (111) crystallographic plane diffraction peak in XRD analysis was determined to be 0.3554 nm, which is notably smaller than the theoretically predicted value of 0.3593 nm [23]. This discrepancy may originate from the significant internal stress present within the T27 sample. The DSC curves of the T16–T21 samples show a two-stage crystallization behavior. The splitting of the first exothermic peak is common in high-Cu-content Fe-based nanocrystalline alloys, which corresponds to the growth of the pre-existing α-Fe crystals and subsequent nucleation and growth of new α-Fe crystals. The second exothermic peak is attributed to the precipitation of the Fe-B phase. The first crystallization peak enthalpy value gradually decreases from 29.81 J/g for T16 to 22.36 J/g and 14.82 J/g for T19 and T21, respectively, indicating the differences in the content of the α-Fe crystalline phase within the as-quenched T16, T19, and T21. Additionally, the decrease in the onset crystallization temperature of the second crystallization peak suggests a deterioration in the stability of the residual amorphous phase. In the as-quenched T27 sample, a large amount of the pre-existing α-Fe and γ-Fe crystalline phases leads to a sharp decrease in the first crystallization peak enthalpy value from 14.82 J/g for T21 to 0.09 J/g for T27. Meanwhile, the splitting of the second crystallization peak in T27 may be attributed to the overlap between the transformation temperature range of the metastable γ-Fe phase and the precipitation of the Fe-B phase. 

**Figure 2:**
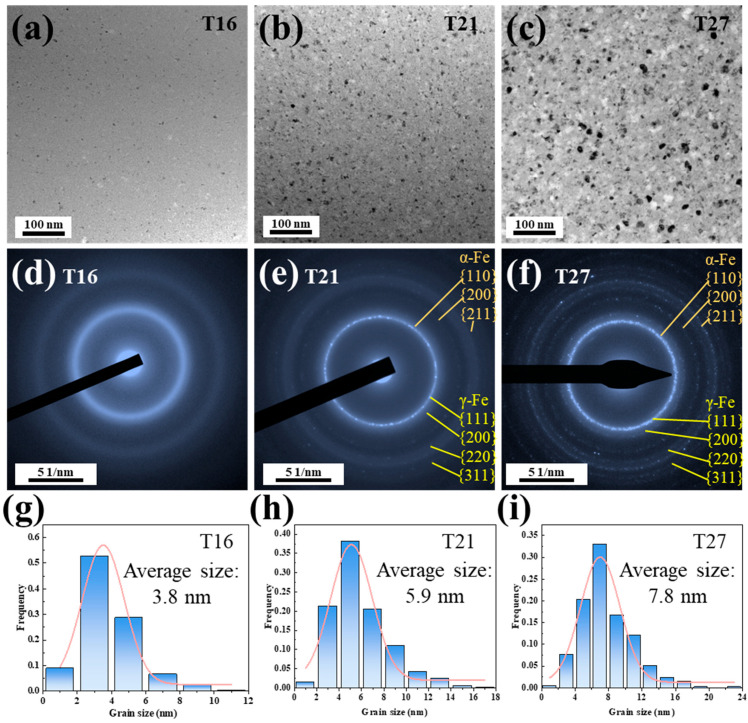
Microstructures near the middle layer of the as-quenched T16, T21, and T27 samples. (**a**–**c**) Bright-field TEM images; (**d**–**f**) selected area electron diffraction patterns; and (**g**–**i**) grain size distribution.

Accounting for the structural heterogeneities present in the thickness direction across all samples, this study conducts a systematic comparative analysis of the structural characteristics at the near middle layer of these samples. The TEM microstructural analysis of the T16, T19, and T27 samples reveals a homogeneous distribution of a certain quantity of subglobular nanocrystals in the amorphous matrix (Figure 2a–c). As the ribbon thickness increases from 16 μm to 21 and 27 μm, the *N*_d_ and *D*_av_ of these nanocrystals increase from 3.02 × 10^23^ m^−3^ and 3.8 nm to 4.85 × 10^23^ m^−3^ and 5.9 nm, 5.13 × 10^23^ m^−3^ and 7.8 nm, respectively, as shown in Figure 2g–i. SAED patterns are employed to analyze the composite structures of these nanocrystals in the T16, T19, and T27 samples. The broadened diffraction halos indicate that the T16 sample is primarily composed of an amorphous structure. The size of the crystalline phase in T16 is so small that it is difficult to be detected in SAED patterns. In Figure 2f, the diffraction rings corresponding to the α-Fe and γ-Fe crystalline phases are detected in T27. In contrast, in T21, the diffraction rings corresponding to the α-Fe crystalline phase were detected, along with some relatively weak diffraction rings associated with the γ-Fe crystalline phase. It should be noted that the content of this part of the γ-Fe crystalline phase is extremely low, resulting in the absence of sharp diffraction peaks in the XRD pattern.

**Figure 3:**
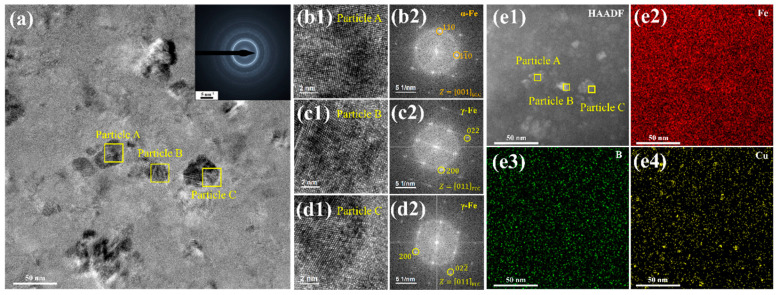
Microstructures near the free side of the as-quenched T27 sample. (**a**) Bright-field TEM image and SAED pattern; (**b1**,**c1**,**d1**) enlarged images of the red square areas in (**a**); (**b2**,**c2**,**d2**) fast Fourier transform images of (**b1**,**c1**,**d1)**, respectively; and (**e1**–**e4**) HAADF-STEM images and mappings of element distribution.

Similar to the structure of the middle layer in the T27 sample, the precipitations of the α-Fe and γ-Fe crystalline phases were also detected in the free side layer in Figure 3a. Due to the difference in cooling rates between the middle layer and the free side layer, the nanocrystals in the free side layer exhibit an irregular polygonal shape with an average size of 8.5 nm. An in-depth structural analysis was conducted on three typical nanocrystalline particles within the red rectangular frame in Figure 3a. The high-resolution TEM (HRTEM) image and fast Fourier transform images presented in Figure 3(b1,b2,c1,c2,d1,d2) confirm the bcc crystalline structure of particle A and the fcc crystalline structure of particles B and C. Furthermore, the HAADF-STEM and EDS mapping results of the same region as in Figure 3a are shown in Figure 3(e1–e4). The results indicate the presence of Fe enrichment corresponding to the positions of the nanoparticles. The distribution of B atoms is relatively homogeneous, while Cu atoms tend to form clusters with a size of approximately 2 nm. Incorporating structural and compositional analyses, it has been determined that two types of bcc α-Fe and fcc γ-Fe nanoparticles coexist in T27.

### 3.2. Crystallization Behavior and Magnetic Properties

**Figure 4:**
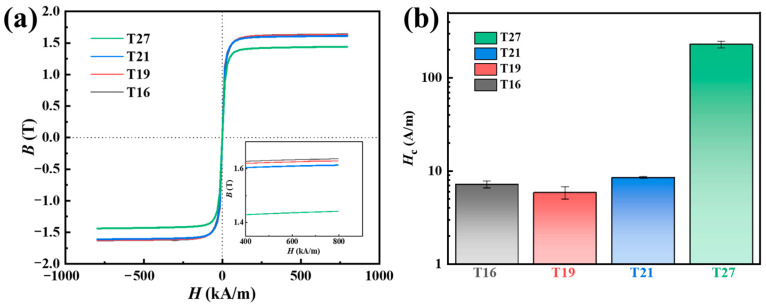
(**a**) *B*_s_ and (**b**) *H*_c_ of the as-quenched Fe_82_B_16.5_Cu_1.5_ alloy ribbons with different thicknesses.

Figure 4a shows the *B*_s_ of the as-quenched Fe_82_B_16.5_Cu_1.5_ alloy ribbons varying with different thicknesses. The result indicates that as the ribbon thickness increases, the *B*_s_ gradually decreases from 1.63 T for T16 to 1.62 T and 1.61 T for T19 and T21. Beyond 21 μm, this reduction accelerates significantly, with *B*_s_ deteriorating to 1.44 T in the T27 sample. For alloys with nanocrystals precipitated in an amorphous matrix, *B*_s_ is determined by the residual amorphous phase and precipitated nanocrystals in terms of *B*_s_ = *B*_sc_*V*_cry_ + *B*_sa_(1 − *V*_cry_), where *B*_sc_ and *B*_sa_ are the saturation flux density of the crystalline phase and amorphous phase, respectively, and *V*_cry_ is the crystallization volume fraction. The *B*_sc_ is commonly higher than the *B*_sa_ [24,25]. However, the samples with increased crystalline phase precipitation demonstrate reduced *B*_s_ as the ribbon thickness escalates. This anomalous phenomenon is primarily attributed to cooling rate variations induced by different thicknesses, which promotes the preferential precipitation of the γ-Fe crystalline phase. The formation of the γ-Fe crystalline phase counteracts the potentially positive effects of the increased nanocrystal population on *B*_s_. Simultaneously, the substantial precipitation of the γ-Fe crystalline phase also gives rise to a remarkable increase in *H*_c_ (228.6 ± 18.7 A/m) for T27. For samples T16, T19, and T21, in which the γ-Fe crystalline phase either does not precipitate or precipitates in extremely small amounts, the *H*_c_ is merely 6–9 A/m.

**Figure 5:**
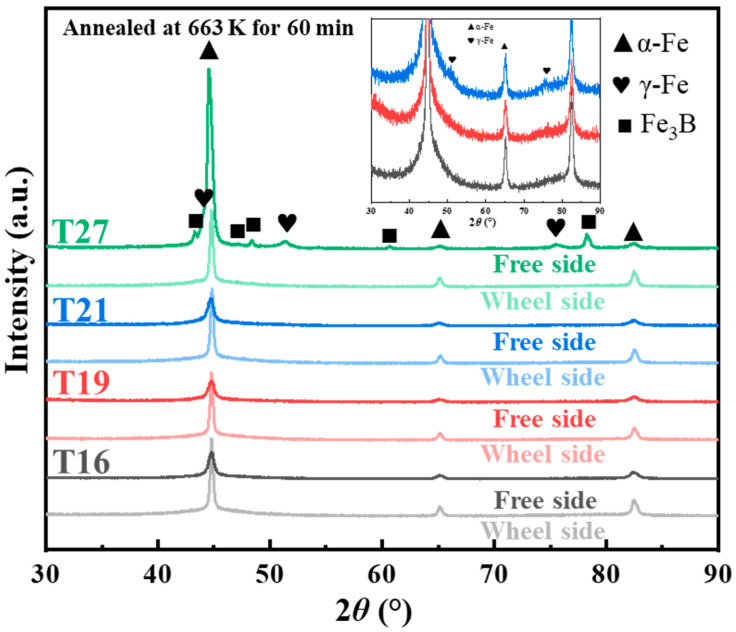
XRD patterns of both surfaces of the annealed T16, T19, T21, and T27 samples. The inset presents an enlarged view of the XRD patterns corresponding to the free side of the annealed T16, T19, and T21 samples.

As is well known, fcc γ-Fe is a metastable phase at room temperature. If it can be transformed into the ferromagnetic bcc α-Fe phase during subsequent annealing, it will have a beneficial effect on magnetic properties. The T16–T27 samples were subjected to prolonged annealing at 663 K for 1 h. The XRD patterns after annealing are shown in Figure 5. On the wheel side, since all samples were in a fully amorphous state in the quenched state, the T16, T19, T21, and T27 samples exhibited identical phase structures after annealing at the same temperature, with partial precipitation of the bcc α-Fe phase. On the free side, the as-quenched state already displayed different phase structures for the T16, T19, T21, and T27 samples due to the different cooling rates. After annealing, both the T16 and T19 samples showed significant precipitation of the α-Fe phase, while the T21 sample exhibited coexistence of the fcc γ-Fe and bcc α-Fe phases. Additionally, the T27 sample revealed a certain amount of the Fe_3_B phase. This indicates that, under the same composition, the structural stability of alloys in different initial states varies, a finding corroborated by the results of the second crystallization peak in DSC analysis. Notably, the fcc γ-Fe phase persisted even after prolonged annealing, which demonstrates that these nanoscale γ-Fe phases exhibit a certain level of thermal stability.

**Figure 6:**
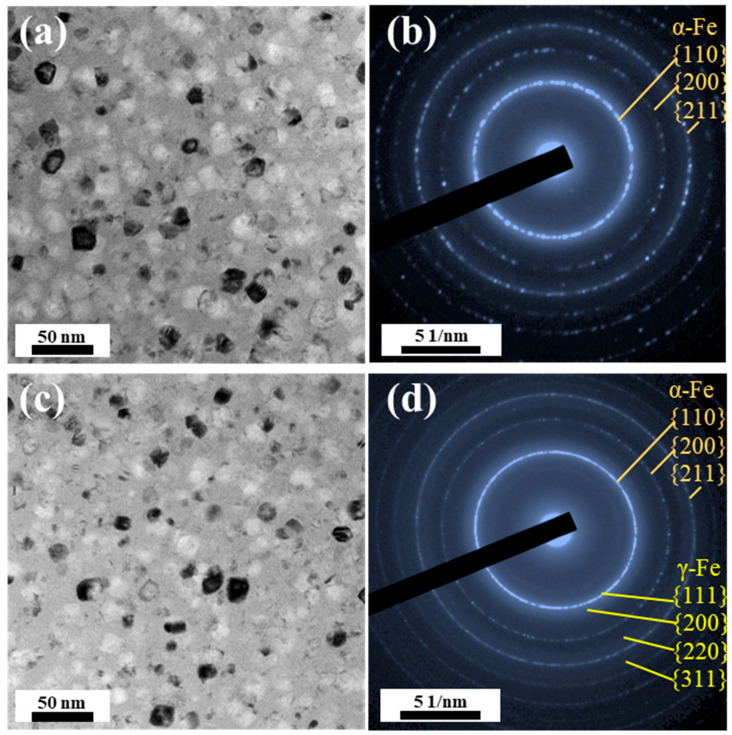
Microstructures near the middle layer of the annealed T16 and T21 samples. (**a**,**c**) Bright-field TEM images; (**b**,**d**) selected area electron diffraction patterns.

The magnetic properties of the annealed samples were characterized. The *B*_s_ for the T16, T19, T21, and T27 samples after annealing were 1.82, 1.76, 1.72, and 1.60 T, respectively, representing increases of 0.19, 0.14, 0.11, and 0.16 T compared to their quenched states. For the T16–T21 samples, the variation in *B*_s_ can be well explained by the differences in the first crystallization peak enthalpy value observed in the DSC curves. Since the amount of the bcc α-Fe phase precipitated in the quenched state differed, T16, which had less of the bcc α-Fe phase in the quenched state, resulted in more newly precipitated α-Fe phase during annealing, achieving the maximum enhancement in *B*_s_. For T27, the significant improvement in *B*_s_ may be attributed to the transformation from the fcc γ-Fe to bcc α-Fe phases. However, the *B*_s_ of annealed T27 was still lower than that of T16, which was partly due to the residual fcc γ-Fe and partly due to the precipitation of some of the Fe_3_B phase. The coercivity of Fe-based nanocrystalline alloys is closely related to their microstructure. Based on the random anisotropy model, when the *D*_av_ value is smaller than the ferromagnetic exchange length (20–40 nm), the magneto-crystalline anisotropy will be averaged out, resulting in a low coercivity. The coercivity is often considered to be proportional to *D*_α-Fe_^6^, where *D*_α-Fe_ denotes the average grain size of α-Fe nanoparticles [5]. Considering the structural differences along the thickness direction of the samples, we separately analyzed the *D*_α-Fe_ values in regions near the free side, near the wheel side, and near the middle layer, as shown in Table 1. The *D*_α-Fe_ values near the free surface and the wheel surface were calculated using the Scherrer formula based on the full width at half-maximum of the α-Fe (110) peak in the XRD results, while the *D*_α-Fe_ value near the middle layer was determined from TEM observations, as shown in Figure 6. For the region near the wheel side of the samples, the T16, T19, T21, and T27 samples exhibited coarse α-Fe nanoparticles with *D*_α-Fe_ values of 34.6, 34.8, 34.2, and 34.2 nm, respectively, whereas, for the region near the free side, the *D*_α-Fe_ values of T16, T19, and T21 are only 15.8, 14.3, and 14.1 nm, respectively. The significant disparity in *D*_α-Fe_ between the free side and the wheel side layer after annealing is attributed to the differences in the quenched-state structure. In the free side layers, the growth of pre-existing α-Fe competes with the subsequently precipitated α-Fe crystalline, resulting in smaller *D*_α-Fe_. In contrast, the wheel side, being in an amorphous state, experiences rapid grain growth of α-Fe crystalline during the annealing process, leading to the formation of a coarse-grained microstructure. Collectively, T21 exhibits smaller α-Fe grain sizes yet higher *H*_c_ compared to T16, which can be primarily attributed to the micro γ-Fe phase precipitation. The *H*_c_ of T27 arises from the precipitation of the γ-Fe phase and Fe_3_B phase, as the formation of these non-ferromagnetic phases can obstruct the displacement of magnetic domain walls [5,14]. By controlling the ribbon thickness to suppress the precipitation of the γ-Fe and Fe_3_B phases, the T16 sample demonstrated an optimal combination of high *B*_s_ (1.82 T) and low *H*_c_ (8.3 A/m). Additionally, it exhibited some thermal stability, enabling prolonged annealing durations.

### 3.3. Formation of the γ-Fe Phase

**Figure 7:**
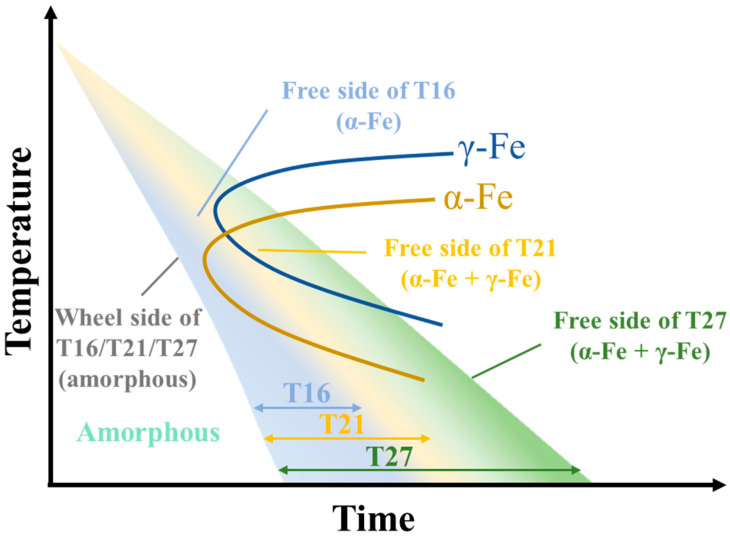
Schematic TTT curves for the quenching process of Fe_82_B_16.5_Cu_1.5_ alloys.

In Fe-based soft magnetic amorphous alloys, the addition of other metallic elements such as Ni can lead to the formation of an fcc (Fe, Ni) phase [26]. Nevertheless, the formation of the fcc γ-Fe phase in the Fe-B-Cu amorphous system remains scarcely reported. The pure bulk fcc γ-Fe phase exists exclusively at elevated temperatures. However, the nanoscale fcc γ-Fe phase can be stabilized at significantly lower temperatures, such as in the form of coherent precipitates within copper matrices or as thin films deposited on copper/silver substrates [27,28,29]. Furthermore, computational studies have revealed that the fcc γ-Fe phase exhibits enhanced thermodynamic stability over the bcc α-Fe phase at the nanoscale [30]. According to previous research, the presence of Cu phases can promote the precipitation of the fcc γ-Fe phase due to the favorable lattice matching between the (111) planes of fcc γ-Fe and fcc Cu [31]. However, in the T27 sample, TEM characterization revealed no evidence of coherent coexistence between the fcc γ-Fe and Cu clusters, indicating that the precipitation mechanism of fcc γ-Fe in T27 may differ from conventional pathways. Based on the Fe-Cu phase diagram, the solid solubility of Cu in the fcc γ-Fe phase is significantly higher than the Cu content in the T27 alloy [32]. Therefore, Cu clusters cannot form prior to the precipitation of fcc γ-Fe. Interestingly, while rapid cooling is generally considered favorable for retaining high-temperature phase constituents, the T27 alloy with a relatively slower cooling rate exhibited a higher volume fraction of retained fcc γ-Fe phase compared to the T21–T27 samples. This apparent paradox suggests a cooling rate-dependent phase selection mechanism. Figure 7 shows the schematic time–temperature–transformation (TTT) curves for the quenching process of the T21–T27 samples. The high thermal conductivity of the copper roller enables the wheel-side surface of the T16–T27 specimens to undergo an ultra-rapid quenching process (cooling rate > 10^9^ K/s), which kinetically suppresses crystallization and stabilizes a fully amorphous structure. For the free side of the T16 sample, the cooling path intersects the α-Fe phase forming zone, leading to the formation of fine α-Fe nanoparticles, while the remaining undercooled liquid directly solidifies into an amorphous matrix. Notably, although the cooling rate at the free side is lower than that at the wheel side interface, it remains sufficiently high (>10^6^ K/s) to kinetically bypass the γ-Fe phase forming zone. When the ribbon thickness increases to 21 μm of the T21 sample, the reduced cooling rate shifts the thermal trajectory to intersect both the γ-Fe and α-Fe phase forming zone boundaries. This results in the precipitation of micro γ-Fe nanoparticles alongside predominant α-Fe nanocrystallites. Further thickening of T27 to 27 μm induces a pronounced overlap of the cooling curve with both the γ-Fe and α-Fe phase domains. Consequently, substantial amounts of γ-Fe and α-Fe nanoparticles precipitate. The diminished cooling rate simultaneously promotes the coarsening of these nanoparticles. The schematic TTT diagram elucidates the origin of phase structure discrepancies in specimens with varying thicknesses. A critical question arises: how can the γ-Fe phase, which is thermodynamically stable only at elevated temperatures, be retained in the quenched microstructure at room temperature? The stabilization of the γ-Fe phase arises from two primary factors. Kinetic suppression occurs as the rapid cooling rates during fabrication inhibit the atomic rearrangement necessary for the γ→α transformation. Simultaneously, nanoscale stabilization emerges due to the increased contribution of interfacial free energy to the total Gibbs free energy with decreasing grain size. The lower interfacial free energy of γ-Fe compared to α-Fe renders its total Gibbs free energy (comprising contributions from both crystallites and interfaces) thermodynamically favorable at the nanoscale, thereby stabilizing the γ-Fe phase [30]. This phase selection mechanism establishes a framework for optimizing high-Cu nanocrystalline alloys by balancing the cooling rate, while simultaneously offering guidance for fabricating high-Cu-content nanocrystalline alloys.

## 4. Conclusions

The investigation of high-Cu-content Fe_82_B_16.5_Cu_1.5_ nanocrystalline alloys revealed the anomalous precipitation of the metastable fcc γ-Fe phase, arising from a phase-selection mechanism under tailored cooling rates. Ribbon thickness modulation (16–27 μm) enabled control of cooling gradients, with thicker ribbons (e.g., 27 μm) stabilizing γ-Fe via kinetic suppression during rapid solidification and nanoscale interfacial energy effects. The γ-Fe phase significantly degraded *B*_s_ and *H*_c_. Optimal soft magnetic properties (*B*_s_ = 1.82 T, *H*_c_ = 8.3 A/m) were achieved in 16 μm-thick ribbons by suppressing γ-Fe and Fe_3_B precipitation. Structural heterogeneity across ribbon layers, driven by cooling rate disparities, led to coarse α-Fe grains on the wheel side and finer nanocrystals on the free side, with γ-Fe stability further validated by its persistence post-annealing. These findings underscore the interplay between cooling kinetics and phase selection, offering structural design guidelines for alloys containing pre-existing α-Fe nanocrystals.

## Figures and Tables

**Table 1:** *D*_av_/*D*_α-Fe_, *B*_s_, and *H*_c_ of annealed Fe_82_B_16.5_Cu_1.5_ alloy ribbons with different thicknesses.

Sample	Phase Composition		*D*_av_/*D*_α-Fe_ (nm)			*B*_s_ (T)	*H*_c_ (A/m)
	Near Wheel	Near Free	Near Wheel	Near Middle	Near Free		
T16	Amorphous + α-Fe	Amorphous + α-Fe	34.6	13.4	15.8	1.82	8.3 ± 0.1
T19	Amorphous + α-Fe	Amorphous + α-Fe	34.8	/	14.3	1.76	13.6 ± 1.1
T21	Amorphous + α-Fe	Amorphous + α-Fe + micro γ-Fe	34.2	10.9	14.1	1.72	38.4 ± 0.6
T27	Amorphous + α-Fe	Amorphous + α-Fe + γ-Fe + Fe_3_B	34.2	/	/	1.60	455.9 ± 2.4

## Data Availability

The original contributions presented in this study are included in the article. Further inquiries can be directed to the corresponding authors.
